# Bridging One Health and Sustainability: The anatomy of a socioethical issue

**DOI:** 10.1093/biosci/biaf008

**Published:** 2025-03-31

**Authors:** Antoine Boudreau LeBlanc, Jérôme Pelletier, Cécile Aenishaenslin, Olivier Beauchet, Manon Boiteux, Michèle Bouchard, Ryoa Chung, Evelyne de Leeuw, Charles Dupras, Xavier Gravend-Tirole, Timothé Poisot, Jean-Philippe Rocheleau, Nicolas Macia, Luc Stafford

**Affiliations:** Institute of Data Valorization, Université de Montréal, McGill University, Montréal, Québec, Canada; Université de Montréal, Saint-Hyacinthe, Québec, Canada; One Health Research Laboratory, Université de Montréal, Saint-Hyacinthe, Québec, Canada; Université de Montréal, Montréal, Québec, Canada; One Health, Université de Montréal, Montréal, Québec, Canada; Department of Environmental, Université de Montréal, Montréal, Québec, Canada; Department of Philosophy, Faculty of Medicine. Centre for Research in Ethics (Centre de Recherche en Éthique), Université de Montréal, Montréal, Québec, Canada; One Urban Health, Université de Montréal, Montréal, Québec, Canada; University of New South Wales Cities Institute, Sydney, Australia; Programme de Bioéthique, School of Public Health in the Department of Social and Preventive Medicine, Université de Montréal, Montréal, Québec, Canada; Université de Montréal, Montréal, Québec, Canada; Université de Montréal, Montréal, Québec, Canada; One Health Research Laboratory, Department of Animal Health, Saint-Hyacinthe College, Saint-Hyacinthe, Québec, Canada; One Health Initiative, Université de Montréal, Montréal, Québec, Canada; One Health Initiative, Université de Montréal, Montréal, Québec, Canada

One Health thrives in several practical areas, such as food safety, antimicrobial resistance, and zoonotic epidemiology. In each, it advocates for a broader scope and addresses complex issues. Its initiative embraces an ever-growing disciplinary inclusiveness and seeks to bridge science and society by including social and human science lenses (Miao et al. 2022, The Lancet 2023, Aguiar et al. 2024). Recognizing the translational gap between theory and practice, there is a growing interest in harmonizing One Health’s lexicon (Boudreau LeBlanc et al. 2022, Aguiar et al. 2024, Ruckert et al. 2024). We must make explicit ethical trade-offs among the values, disciplines, and knowledge inherent in One Health’s operationalization (Li 2020) while acknowledging the associated stakes. This Viewpoint outlines the practical and ethical challenge of negotiating daily priorities between health impetus and the ecological cascade of effects for concerned scientists, professionals, and citizens.

One Health, planetary health, and ecohealth are integrated approaches arising from diverse academic and sectoral settings in response to growing civil, political, and scientific concerns regarding well-being and the environment. Despite their distinct foundations leading to their own set of terminologies, priorities, and methodologies, they each pursue similar goals (Boudreau LeBlanc 2023). They all seek to translate the understanding of concepts from the philosophy of complexity, such as interconnectivity and interdependence, into the reality of species, societies, and other entities (Lerner and Berg 2017), including the complexity of sciences, calling for greater integration across disciplines, sectors, localities, and generations (Boudreau LeBlanc 2023). Initiated in the mid-1980s, the biomedical shift toward health promotion has sparked political interest in the health–environment relationship (de Leeuw et al. 2024). This exposed health to the multifaceted, complex, and escalating environmental challenges highlighted by concerned scholars, such as Aldo Leopold and Rachel Carson, alongside a growing body of literature today (Norton 2017, Millstein 2018, Cena and Labra 2024).

Instead of promoting a confrontational perspective, we encourage a constructive dialogue among approaches, using our One Health understanding as a catalyst for engaging conversation (Boudreau LeBlanc 2023). Although One Health scholars acknowledge the wicked problems inherent in systems thinking and managing complex issues, they struggle to untie ethical dilemmas in practice (Waltner-Toews 2017). A political force drives One Health internationally by developing its concept in recommendations (Gruetzmacher et al. 2021). Initially led by public health veterinarians, the COVID-19 pandemic has prompted reflections across practices, sectors, and disciplines within universities and beyond. More recently, the 2021 international Quadripartite One Health definition has emphasized “building interdisciplinary bridges to health” (Zinsstag et al. 2011, Destoumieux-Garzón et al. 2018, WHO et al. 2022, Lefrançois et al. 2023). However, fostering “interdisciplinary bridges [beyond] health”—that is, engaging with sustainability scholars—remains a major challenge (Boudreau LeBlanc et al. 2022).

## One Health as a negotiating environment

The challenge arises from the narrow meaning still attributed to interdisciplinarity. The architecture of One Health’s integrative and multifaceted operationalization requires substantial theoretically driven efforts and a transcendence beyond the political sphere framed by the Quadripartite definition (Norton 2000, Odenbaugh 2016, Rademacher et al. 2019), which still overlooks the Convention on Biological Diversity. If critical thinking stands implicitly in its definition, we should not underestimate the required rational work (Antoine-Moussiaux et al. 2019). Conceptualization (e.g., integrating, unifying, sustaining, balancing, recognizing, or mobilizing, as used in the definition) demands more than just goodwill (Ehling-Schulz et al. 2024). An One Health textbook should make explicit the pressing need to study contexts, facilitatory actors, actionable leverages, and points of passage that deepen tools, training, and competencies to help its professionals negotiate values and perspectives in the field (Boudreau LeBlanc 2023).

As stated in the 2022–2026 One Health joint action plan, “One Health coordination” implies to “address issues *holistically*; [to] deal with complexity and ambiguity; [to] negotiate trade-offs and identify win–win solutions; and [to] agree on priorities, funds, and collective actions (including monitoring and evaluation)” (WHO et al. 2022). However, it does not acknowledge the challenge of conceptualizing the know-how needed to manage local trade-offs within a broader organizational thinking: “act local; think global” (Rock et al. 2009, Boudreau LeBlanc et al. 2022, de Maack et al. 2024). For example, a considerable burden falls on the shoulders of professionals who may feel alone in negotiating the compromise of prescribing antibiotics to sick patients, contextualized within the dynamics of pharmaceutics and the planetary ecological cascades involving the genesis of microbial resistance, chemical by-products and contaminants, or carbon emissions (Boudreau LeBlanc et al. 2022).

Political discourses tend to prioritize emblematic species or landscapes over the protection of ecosystems and their inhabitants, focusing on either useful or dangerous species and ecological functioning (Tsing 2009, Moreno-Mateos et al. 2015, Rademacher et al. 2019). In contrast, pathogen control and management are seen as acceptable and often vital for improving human health and sustaining agriculture (Shurson et al. 2022). For instance, the rationale for vector reduction is grounded in its measurable impact, which can either strengthen or destabilize ecological resilience (Procopio et al. 2024). However, the complexity arises from the discourses on ecology, which range from an anthropo- or pluri- to acentric focus (Latour 1996, Kimmerer 2002, Blue and Rock 2011, Rademacher et al. 2019, Boudreau LeBlanc 2023). Political ecology centers on a logic of prioritization, mainly the dilemma opposing human goals and habitat functioning. When a trade-off emerges, we must prevent long-term compromise to the environment, because this increases uncertainties and intensifies risks (Rademacher 2015, Boudreau LeBlanc et al. 2022, Aguiar et al. 2024, de Maack et al. 2024).

Such an ambitious One Health paradigm is complex to articulate in practice, because it leads to constant ethical negotiation, trade-offs, and compromise justifications. An ethical dilemma arises when we recognize that all foreseeable action paths lead to harm, but a decision and a structured course of action (a norm) must still be systematized (Beever and Morar 2019, Morar and Bohannan 2019). With its anthropocentric optimism (or positivism) about health, One Health is commendable in envisioning better futures but fails to recognize the inherent consequences of any trade-off in practice. Therefore, studies on negotiation and philosophical investigations should be integrated into One Health research and its education curriculum (Antoine-Moussiaux et al. 2019, Laing et al. 2023).

## Beyond existential risks in health

Ethics scholars increasingly highlight the existential stakes involved (Morar and Bohannan 2019). Human activities tend to promote short-term individual flourishing over ecosystem resilience, which reduces the stability of the habitat conditions for living beings (Potter 1988, Beever and Morar 2019). This decreased stability affects long-term planning, as is now understood, because of climate changes and accentuates inequity because of the scarcity and uneven distribution of resources (Zhang et al. 2024). For example, any health and biodiversity trade-offs while operationalizing One Health should be approached through a reflexive, collaborative, and adaptive effort (Boudreau LeBlanc et al. 2022, de Maack et al. 2024). Therefore, we need an open and constructive forum across sectors and disciplines focused on health and biodiversity while challenging this constructed dichotomy (Dietz and Stern 1998, Romanelli et al. 2014, Boudreau LeBlanc 2023). Addressing this dilemma, sometimes biodiversity should prevail, and other times, health should be prioritized. Ultimately, One Health is doomed to fail if one systematically overshadows the other.

This Viewpoint underlines the urgent need for translational studies in One Health. It means applying a capacity for philosophical negotiation to ethically address trade-offs (de Maack et al. 2024). This entails ethics analysis to unpack dilemmas, deepen dialogues, and generate safe spaces for thinking, planning, and evaluating decisions (Boudreau LeBlanc et al. 2022)—ultimately leading to systematized actions in our complex, intertwined world (Zhang et al. 2024). Drawing on a long-lasting involvement in One Health, both within its community and through its literature, we have observed an emerging political discourse permeating the scientific community. Exercising power, work teams, and resources without recognizing ethical dilemmas, such as the one opposing health and biodiversity, necessitates sustained, individualized, and critical reflexivity (Boudreau LeBlanc 2023). We aim to raise awareness of this trend, which hinders the operationalization of One Health, especially when the community calls for an ever more inclusive *one* (Rodriguez 2024).

Increasing awareness of the asymmetrical privileges in medicine and ecology, as is reflected in political and administrative discourses and economic power, will deepen our understanding of the social dynamics within the One Health community (Boudreau LeBlanc et al. 2022). Medicine typically receives greater institutional support and public funding. Meanwhile, ecology struggles in advocating for its representation despite several Earth Summits and the formation of numerous international committees, programs, and initiatives focused on biodiversity (Legagneux et al. 2018, Pascual et al. 2022). Human health relies on numerous environmental attributes because the sustainability or degradation of habitats delineates existential risks and threats, for both humans and nonhumans.

Definition is a source of power (Ålund 1988, Zarefsky 2014, Cisney and Morar 2015, Skerrat 2016, Boudreau LeBlanc et al. 2022). We must recognize the limits of the reductionism inherent in science, because a mechanistic, technical understanding creates a sense of biological normativity (Giroux 2010). Scholars in the humanities have long emphasized that integrating knowledge implies prioritizing concepts that confer political power, leading to practical implications for health (Kimmerer 2012). But all power comes with its biases, flaws, and assumptions (Kimmerer 2002). In this way, One Health should evolve into a reflexive autocriticism that includes a broader range of disciplines (Boudreau LeBlanc 2023). This means opening the curriculum to the human, subjective aspects of natural sciences—the scientists and institutions behind the One Health approach, techniques, and interventions.

## Author Biography

Antoine Boudreau LeBlanc (antoine.boudreau.leblanc@umontreal.ca) is a postdoctoral researcher in innovation ethics funded by the Institute of Data Valorization, at the Université de Montréal and McGill University, in Montréal, Québec, Canada. Jérôme Pelletier (jerome.pelletier.1@umontreal.ca) is a postdoctoral researcher in epidemiology at the Université de Montréal, in Saint-Hyacinthe, Québec, Canada. Cécile Aenishaenslin is a professor in veterinary epidemiology, public health and intervention research, and codirector of the One Health Research Laboratory at the Université de Montréal, in Saint-Hyacinthe, Québec, Canada. Olivier Beauchet is a professor and clinician-researcher, neurologist, and geriatrician at the Université de Montréal, in Montréal, Québec, Canada. Manon Boiteux is a PhD student in One Health at the Université de Montréal, in Montréal, Québec, Canada. Michèle Bouchard is a professor in the Department of Environmental and Workplace Health and vice dean for research (One Health aspect) at the Université de Montréal, Montréal, Québec, Canada. Evelyne de Leeuw is a professor of public health, cities and policies and the Canada Excellence Research Chair in One Urban Health at the Université de Montréal, in Montréal, Québec, Canada, and is also a professor at the University of New South Wales Cities Institute, in Sydney, Australia. Ryoa Chung is a professor in the Department of Philosophy and also teaches medical ethics at the Faculty of Medicine and codirector of the Centre for Research in Ethics (Centre de Recherche en Éthique) at the Université de Montréal, in Montréal, Québec, Canada. Charles Dupras, is professor of ethics and director of the Programme de Bioéthique at the School of Public Health in the Department of Social and Preventive Medicine at the Université de Montréal, in Montréal, Québec, Canada. Xavier Gravend-Tirole is a professor of ethics and spirituality at the Université de Montréal, in Montréal, Québec, Canada. Timothée Poisot is a professor of computational ecology at the Université de Montréal, in Montréal, Québec, Canada. Jean-Philippe Rocheleau is a professor in epidemiology, codirector of the One Health Research Laboratory and a professor and coordinator of the Department of Animal Health at Saint-Hyacinthe College, in Saint-Hyacinthe, Québec, Canada. Nicolas Macia is the senior coordinator of the strategic institutional project for the One Health Initiative at the Université de Montréal, in Montréal, Québec, Canada. Luc Stafford is a professor of physics and holds the Canada Research Chair in the Physics of Highly Reactive Plasmas and is also deputy vice rector of research and codirector of the One Health Initiative at the Université de Montréal, in Montréal, Québec, Canada.

## References

[bib1] Aguiar R, Keil R, Wiktorowicz M. 2024. The urban political ecology of antimicrobial resistance: A critical lens on integrative governance. Social Science and Medicine 348: 116689. 10.1016/j.socscimed.2024.116689 38564956

[bib2] Ålund A. 1988. The power of definitions: Immigrant women and problem-centered ideologies. Migration: A European Journal of International Migration and Ethnic Relation 4: 37–55.

[bib3] Antoine-Moussiaux N, Janssens de Bisthoven L, Leyens S, Assmuth T, Keune H, Jakob Z, Hugé J, Vanhove MPM. 2019. The good, the bad and the ugly: Framing debates on nature in a one Health community. Sustainability Science 14: 1729–1738. 10.1007/s11625-019-00674-z PMC7088772 32215109

[bib4] Beever J, Morar N. 2019. The epistemic and ethical onus of “One Health”. Bioethics 33: 185–194. 10.1111/bioe.12522 30341916

[bib5] Blue G, Rock M. 2011. Trans-biopolitics: Complexity in interspecies relations. Health: An Interdisciplinary Journal for the Social Study of Health, Illness, and Medicine 15: 353–368. 10.1177/1363459310376299 21169200

[bib7] Boudreau LeBlanc A. 2023. Building the bioethics tools of a community council to the future: The ecosystemic gap. Humanities and Social Sciences Communications 10: 562.

[bib6] Boudreau LeBlanc A, Williams-Jones B, Aenishaenslin C. 2022. Bio-ethics and one health: A case study approach to building reflexive governance. Frontiers in Public Health 10: 274–345. 10.3389/fpubh.2022.648593 PMC8971560 35372246

[bib8] Cena H, Labra M. 2024. Biodiversity and planetary health: A call for integrated action. Lancet 403: 1985–1986. 10.1016/S0140-6736(24)00292-7 38762319

[bib9] Cisney VW, Morar N. 2015. Introduction: Why biopower? Why now? Pages 1–25 in Cisney VW, Morar N, eds. Biopower : Foucault and Beyond. University of Chicago Press.

[bib10] de Leeuw E, Kickbusch I, Rüegg SR. 2024. A health promotion perspective on One Health. Canadian Journal of Public Health 115: 271–275. 10.17269/s41997-024-00872-y PMC11006651 38478217

[bib11] de Maack V, Tubeuf S, Desterbecq C, Dupras C. 2024. Beyond advocacy: Human health, the environment, and tradeoff ethics. American Journal of Bioethics 24: 50–52. 10.1080/15265161.2024.2303164 38394010

[bib12] Destoumieux-Garzón D et al. 2018. The One Health concept: 10 years old and a long road ahead. Frontiers in Veterinary Science 5: 1–13. 10.3389/fvets.2018.00014 PMC5816263 29484301

[bib13] Dietz T, Stern PC. 1998. Science, values, and biodiversity. BioScience 48: 441–444.

[bib14] Ehling-Schulz M et al. 2024. Risk negotiation: A framework for One Health risk analysis. Bulletin of the World Health Organization 102: 453–456. 10.2471/BLT.23.290672 PMC11132153 38812798

[bib16] Giroux É. 2010. Après Canguilhem : Définir la Santé et la Maladie. Presses Universitaires de France.

[bib17] Gruetzmacher K et al. 2021. The Berlin principles on one health: Bridging global health and conservation. Science of the Total Environment 764: 142919. 10.1016/j.scitotenv.2020.142919 PMC7550087 33097250

[bib18] Kimmerer RW. 2002. Weaving traditional ecological knowledge into biological education: A call to action. BioScience 52: 432–438.

[bib19] Kimmerer RW. 2012. Searching for synergy: Integrating traditional and scientific ecological knowledge in environmental science education. Journal of Environmental Studies and Sciences 2: 317–323.

[bib20] Laing G et al. 2023. Advancing One Health: Updated Core Competencies. CABI One Health.

[bib44] Lancet . 2023. One Health: a Call for Ecological Equity. Lancet 401: P169. 10.1016/S0140-6736(23)00090-9 36681403

[bib21] Latour B. 1996. On interobjectivity. Mind, Culture, and Activity 3: 228–245.

[bib22] Lefrançois T, Malvy D, Atlani-Duault L, Benamouzig D, Druais P-L, Yazdanpanah Y, Delfraissy J-F, Lina B. 2023. After 2 years of the COVID-19 pandemic, translating one Health into action is urgent. Lancet 401: 789–794. 10.1016/S0140-6736(22)01840-2 PMC9595398 36302392

[bib23] Legagneux P et al. 2018. Our house is burning: Discrepancy in climate change vs. biodiversity coverage in the Media as compared to scientific literature. Frontiers in Ecology and Evolution 5: 175.

[bib24] Lerner H, Berg C. 2017. A comparison of three holistic approaches to health: One health, EcoHealth, and planetary health. Frontiers in Veterinary Science 4: 163. 10.3389/fvets.2017.00163 PMC5649127 29085825

[bib25] Li BV. 2020. Creating synergy between biodiversity conservation and human health: One Health. Biodiversity Science 28: 596–605.

[bib26] Miao L, Li H, Ding W, Lu S, Pan S, Guo X, Zhou X, Wang D. 2022. Research priorities on One Health: A bibliometric analysis. Frontiers in Public Health 10: 889854. 10.3389/fpubh.2022.889854 PMC9194370 35712284

[bib27] Millstein RL. 2018. Understanding Leopold's concept of “interdependence” for environmental ethics and conservation biology. Philosophy of Science 85: 1127–1139.

[bib28] Morar N, Bohannan BJM. 2019. The conceptual ecology of the human microbiome. Quarterly Review of Biology 94: 149–175.

[bib29] Moreno-Mateos D, Maris V, Béchet A, Curran M. 2015. The true loss caused by biodiversity offsets. Biological Conservation 192: 552–559.

[bib30] Norton BG. 2000. Biodiversity and environmental values: In search of a universal earth ethic. Biodiversity and Conservation 9: 1029–1044.

[bib31] Norton BG. 2017. A situational understanding of environmental values and evaluation. Ecological Economics 138: 242–248.

[bib32] Odenbaugh J. 2016. Protecting biodiversity and moral psychology; or why philosophers are asking the wrong questions. Pages 193–211 in Garson J, Plutynski A, Sarkar S, eds. The Routledge Handbook of Philosophy of Biodiversity. Routledge.

[bib33] Pascual U et al. 2022. Governing for transformative change across the biodiversity–climate–society nexus. BioScience 72: 684–704.

[bib34] Potter VR . 1988. Global Bioethics: Building on the Leopold Legacy. Michigan State University Press.

[bib35] Procopio AC, Colletta S, Laratta E, Mellace M, Tilocca B, Ceniti C, Urbani A, Roncada P. 2024. Integrated one health strategies in Dengue. One Health 18: 100684. 10.1016/j.onehlt.2024.100684 PMC11247296 39010969

[bib37] Rademacher A. 2015. Urban Political Ecology. Annual Review of Anthropology 44: 137–152.

[bib36] Rademacher A, Cadenasso ML, Pickett STA. 2019. From feedbacks to coproduction: Toward an integrated conceptual framework for urban ecosystems. Urban Ecosystems 22: 65–76.

[bib38] Rock M, Buntain BJ, Hatfield JM, Hallgrímsson B. 2009. Animal–human connections, “One Health,” and the syndemic approach to prevention. Social Science and Medicine 68: 991–995. 10.1016/j.socscimed.2008.12.047 19157669

[bib39] Rodriguez J. 2024. One Health ethics and the ethics of zoonoses: A silent call for global action. Veterinary Sciences 11: 394. 10.3390/vetsci11090394 PMC11435914 39330773

[bib40] Romanelli C, Cooper HD, Dias S. 2014. The integration of biodiversity into one health. Revue Scientifique et Technique de L'oie 33: 487–496. 10.20506/rst.33.2.2291 25707179

[bib41] Ruckert A, Harris F, Aenishaenslin C, Aguiar R, Boudreau-LeBlanc A, Pedro Carmo L, Labonté R, Lambraki I, Parmley EJ, Wiktorowicz ME. 2024. One health governance principles for AMR surveillance: A scoping review and conceptual framework. Research Directions: One Health 2: e4.

[bib42] Shurson GC, Urriola PE, Ligt JLG. 2022. Can we effectively manage parasites, prions, and pathogens in the global feed industry to achieve one health? Transboundary and Emerging Diseases 69: 4–30. 10.1111/tbed.14205 34171167

[bib43] Skerrat S. 2016. The power of rhetoric in re-shaping health and social care: Implications for the under-served. Sociologia e Politiche Sociali 4: 9–28.

[bib45] Tsing A. 2009. Beyond economic and ecological standardisation. Australian Journal of Anthropology 20: 347–368.

[bib46] Waltner-Toews D. 2017. Zoonoses, One Health, and complexity: Wicked problems and constructive conflict. Philosophical Transactions of the Royal Society B 372: 20160171. 10.1098/rstb.2016.0171 PMC5468696 28584179

[bib15] [WHO, et al.] World Health Organization, Food and Agriculture Organization of the United Nations, World Organisation for Animal Health, United Nations Environment Programme . 2022. One Health Joint Plan of Action (2022–2026): Working Together for the Health of Humans, Animals, Plants and the Environment. WHO.

[bib47] Zarefsky D. 2014. Presidential rhetoric and the power of definition. Pages 225–238 in Zarefsky D, ed. Rhetorical Perspectives on Argumentation: Selected Essays by David Zarefsky. Springer.

[bib48] Zhang L, Liu S, Guo W, Lv C, Liu X. 2024. Addressing biodiversity conservation, disease surveillance, and public health interventions through Hne Health approach in Hainan's tropical rainforest. One Health Advances 2: 1–17.

[bib49] Zinsstag J, Schelling E, Waltner-Toews D, Tanner M. 2011. From “one medicine” to “One Health” and systemic approaches to health and well-being. Preventive Veterinary Medicine 101: 148–156. 10.1016/j.prevetmed.2010.07.003 PMC3145159 20832879

